# Linking laboratory findings to field fertility: a comparative study of frozen semen from dairy and beef bulls

**DOI:** 10.3389/fvets.2026.1794630

**Published:** 2026-02-27

**Authors:** Alper Kocyigit, Huseyin Un, Cumali Kaya, Burcu Esin, Merve Deniz Tanrıkulu, Mesut Cevik

**Affiliations:** Department of Reproduction and Artificial Insemination, Faculty of Veterinary Medicine, Ondokuz Mayis University, Samsun, Türkiye

**Keywords:** artificial insemination, dairy and beef bulls, field fertility, motility, sperm kinematics

## Abstract

Artificial insemination in cattle relies on laboratory semen evaluation, yet field fertility often diverges from laboratory-based expectations. This study compared spermatological and computer-assisted sperm analysis (CASA) kinematic traits of commercially frozen–thawed semen from dairy and beef bulls and examined their associations with pregnancy outcome under commercial conditions. Frozen semen from 11 bulls (beef: Angus, *n* = 5; dairy: Holstein, *n* = 6) was obtained through the Amasya Cattle Breeders’ Association; six straws from the same batch per bull were analysed and averaged at the bull level. After thawing (37 °C, 30 s), motility, sperm concentration, and kinematic parameters were assessed using CASA under standardized settings. Field inseminations were performed by a single veterinarian in healthy multiparous cows showing spontaneous estrus. A total of 413 inseminations were conducted (beef semen: 194; dairy semen: 219); pregnancy diagnosis was available for 409 inseminations at day 60. Mean daily milk yield (days 5–305) was derived from association records (beef females, *n* = 194; dairy females, *n* = 219). Semen from beef bulls showed higher total motility (83.59 ± 2.08% vs. 56.24 ± 2.42%; *p* < 0.0001) and higher progressive motility (58.54 ± 3.01% vs. 48.25 ± 9.57%; *p* = 0.0475) than semen from dairy bulls. Dairy bulls had higher STR and LIN and lower ALH, whereas velocity descriptors differed modestly and did not show consistent between-group separation. Mean bull-level pregnancy rate was numerically higher for beef than dairy bulls (68.06% vs. 63.71%), without a significant between-group difference at the insemination level. Daily milk yield differed markedly between female groups (18.47 ± 0.38 vs. 6.21 ± 0.19 kg/d; *p* < 0.001). Within-group Pearson correlations indicated that motility was positively associated with pregnancy rate in both production types, while several kinematic descriptors exhibited production-type-specific correlation directions. These findings suggest that production context may modulate how CASA phenotypes relate to field fertility, supporting cautious interpretation of single-parameter semen metrics in commercial AI systems.

## 1 Introduction

Artificial insemination (AI) is a central tool for genetic improvement in contemporary cattle production, enabling the rapid dissemination of genetically high-merit germplasm through the globally standardized infrastructure underpinning frozen bull semen production and distribution (1). Despite advances in semen processing, cryopreservation, extender design, and quality control, pregnancy rates following AI still vary markedly across farms and seasons, and also among bulls and technicians (2–4). Epidemiological data further suggest that favourable laboratory-based semen quality metrics do not consistently translate into high field fertility, a pattern that may reflect the combined influence of environmental conditions and sire-specific biological differences that are incompletely captured by routine assays (5, 6).

Because dairy- and beef-oriented cattle populations have been subject to long term selection under differing production goals, they may also differ in reproductive phenotypes associated with sperm structure, metabolic traits, and resilience to cryoinjury (7, 8). Comparative studies suggest that production type can be reflected in post-thaw sperm quality profiles. In some cohorts, dairy bulls have tended to show higher plasma membrane integrity and a greater proportion of morphologically normal sperm, alongside higher indicators of mitochondrial respiratory activity and lower DNA fragmentation indices than beef bulls (8, 9). Complementing these univariate contrasts, multivariate approaches further indicate that bull type can contribute measurably to the overall semen quality signature beyond standardized processing conditions (10, 11). It is also important to note that production type often co-varies with breed composition and management context, which may jointly shape observed differences under field conditions.

These observations are compatible with the hypothesis that biology associated with breed or production type may modulate cryotolerance through differences in membrane composition and stability, mitochondrial function, and susceptibility to oxidative stress, factors repeatedly implicated in variation in sperm freezability among bulls (5, 12). Classical comparisons between beef-type and dairy-type bulls have also reported lower motility, viability, and normal morphology, together with a higher prevalence of specific primary sperm abnormalities in certain beef breeds, patterns that may suggest differences in spermatogenic efficiency and in sensitivity to physiological or environmental stressors (7, 9). Taken together, the available evidence supports the view that semen from dairy versus beef bulls may diverge not only in kinematic behaviour but also in functional attributes relevant to fertilizing potential, including membrane and acrosomal status, mitochondrial competence, and chromatin integrity, which may contribute to variability in field fertility even when semen is produced under standardized laboratory protocols (5, 6).

Computer-assisted sperm analysis (CASA) is widely used for objective semen evaluation, enabling quantitative assessment of sperm kinematics such as average path velocity (VAP), straight-line velocity (VSL), and curvilinear velocity (VCL), together with related descriptors including linearity (LIN), amplitude of lateral head displacement (ALH), and beat-cross frequency (BCF). Relative to conventional subjective microscopic assessment, CASA can improve measurement objectivity and within-laboratory repeatability when sampling conditions and instrument settings are carefully standardized, although comparability across systems and settings can still vary (13). Although some studies have reported significant associations between CASA derived kinematic parameters and fertility outcomes (3), others highlight the multifactorial determinants of reproductive success and suggest that individual kinematic variables, considered in isolation, offer limited predictive utility under commercial field conditions (6). This has motivated the use of multivariate analytical strategies and sperm subpopulation profiling as potentially more informative frameworks for linking motion phenotypes to fertility-relevant function.

Much of the existing literature has examined a single production type, often dairy cattle, or has evaluated semen primarily through *in vitro* assessments performed under controlled laboratory conditions. Consequently, it remains unclear whether production type associated differences in sperm kinematics and related functional attributes translate into meaningful variation in reproductive performance in commercial field settings. Field studies that jointly consider production type, detailed kinematic semen parameters, and pregnancy outcomes while maintaining comparable management, environmental exposure, and insemination procedures appear limited, despite their practical importance for AI program refinement. This evidence gap complicates the generalization of laboratory-derived semen quality indicators and reduces confidence in their use for fertility prediction across heterogeneous bull populations. It was hypothesized that frozen–thawed semen from dairy and beef bulls exhibits distinct post-thaw quality and kinematic profiles and that these differences are associated with post-AI pregnancy outcomes. The objective of this study was to compare post-thaw spermatological and CASA-derived kinematic traits of frozen semen from Holstein (dairy) and Angus (beef) bulls and to explore whether between-bull variation in these laboratory traits is associated with field pregnancy outcomes within the production systems in which the semen was used. As an observational study conducted under routine commercial conditions, this work aims to explore these relationships rather than establish causal attribution between sire production type and pregnancy success. This study aims to exploratorily evaluate the associations between spermatological parameters measured under commercial field conditions and fertility outcomes.

## 2 Materials and methods

### 2.1 Semen samples

This study utilized commercially frozen bovine semen obtained from the Amasya Cattle Breeders’ Association and produced by STgenetics (Texas, United States). Semen from a total of 11 bulls was included, comprising five Angus bulls (beef type) and six Holstein bulls (dairy type). All semen straws were processed at the same production centre under routine commercial protocols and stored in liquid nitrogen until use.

### 2.2 Spermatological analysis

Analyses were conducted using six straws originating from the same production batch for each bull, and the obtained values were averaged on a per-bull basis. Prior to evaluation, semen straws were thawed in a water bath at 37 °C for 30 s. Sperm motility, concentration, and kinematic characteristics were assessed with a Computer-Assisted Sperm Analysis (CASA) system (Sperm Class Analyzer (SCA), Version 6.5.0.91, Microptic, Barcelona, Spain). Evaluations were carried out using a phase-contrast microscope (Eclipse, Nikon, Tokyo, Japan) fitted with a 10 × objective and a high-speed camera operating at 60 frames per second, while the microscope stage temperature was maintained at 37 °C. Sperm images were acquired using a high-frame-rate Basler digital camera provided by the CASA system manufacturer (Microptic S. L., Barcelona, Spain) and mounted on the phase-contrast microscope. CASA configuration parameters were standardized for ruminant spermatozoa as follows: frame rate of 25 Hz, acquisition duration of 1 s, minimum contrast of 80, minimum cell size of 4 μm^2^, VAP threshold of 20 μm/s, and VSL threshold of 10 μm/s. The evaluated parameters included total motility (%), progressive motility (%), VSL, VAP, VCL, ALH, BCF, STR, LIN, and WOB. For each sample, at least 200 spermatozoa, randomly chosen from a minimum of five microscopic fields, were analysed.

### 2.3 Artificial insemination procedures

**Figure 1:**
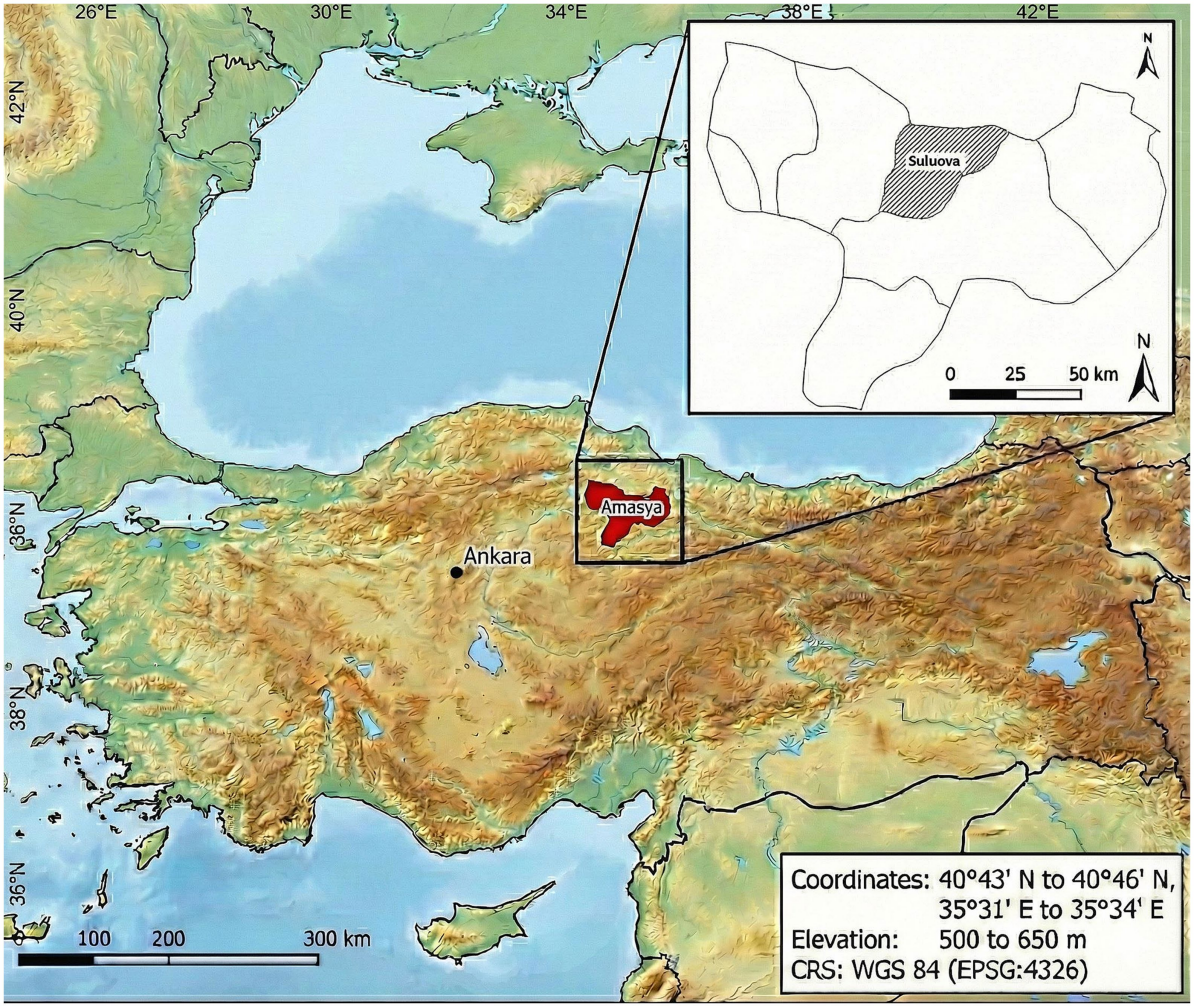
The geographical location of the study area.

Field artificial inseminations were performed in small and medium-scale cattle farms located in the Suluova district of Amasya, Türkiye (Figure 1). These farms had similar management, feeding, and environmental conditions. Estrus detection was initiated by farmer notifications and subsequently confirmed by a veterinarian. All inseminations were performed by the same licensed veterinarian using the recto-vaginal technique in clinically healthy, multiparous cows exhibiting spontaneous estrus. Within each production system, semen straws were randomly selected from the pool of bulls compatible with the female’s production type and breed.

A total of 413 inseminations were conducted, including 194 inseminations with semen from beef-type bulls and 219 inseminations with semen from dairy-type bulls. Pregnancy rate calculations were based exclusively on outcomes following the first insemination; results from second, third, and subsequent inseminations in the same animal were not included in the analysis. Pregnancy diagnosis was performed in 409 females on day 60 after insemination using transrectal palpation and ultrasonography. Four cows were excluded from the final analysis due to being culled or sold before the pregnancy diagnosis. Pregnancy outcomes were recorded for further statistical evaluation.

### 2.4 Milk yield assessment of inseminated females

Milk yield data were obtained from regularly recorded breeding association records. In both production systems, cows were milked twice daily. For each inseminated female, daily milk yield was calculated based on recorded production values between days 5 and 305 of the most recent lactation, corresponding to the standard lactation period. Mean daily milk yield was then calculated for each production group and used for comparative analyses between dairy and beef females.

### 2.5 Statistical analysis

Statistical analyses were performed using IBM SPSS Statistics for Windows, Version 25.0 (IBM Corp., Armonk, NY, USA). Normality of continuous variables was assessed using the Shapiro–Wilk test and homogeneity of variances using Levene’s test where applicable; when the homogeneity assumption was violated, Welch’s *t*-test was applied. Descriptive statistics for spermatological and kinematic parameters are presented as mean ± standard deviation (SD).

An *a priori* power analysis was conducted using G*Power (v3.1.9.7; Heinrich-Heine-Universität Düsseldorf, Germany) with power = 0.80 and *α* = 0.05. The planned sample size was sufficient to detect a 15-percentage point difference in pregnancy rates between groups. In total, 413 inseminations were performed (beef semen: 194; dairy semen: 219); pregnancy outcome was available for 409 inseminations after exclusion of four cases without a valid pregnancy diagnosis (beef: 190; dairy: 219). With the achieved sample size (*n* = 409), the estimated power to detect a 15-percentage point difference was approximately 0.87, whereas for a 10-percentage point difference it was approximately 0.53.

Bull usage was quantified as the number of inseminations per bull within each production type, and between-group differences in bull-level insemination counts were evaluated using the same assumption-based framework described above.

Milk yield was analysed at the female level using each female’s mean daily yield (days 5–305) as a single summary measure. Between-group differences (dairy vs. beef females) in mean daily milk yield were tested using an independent samples t test when distributional assumptions were met (Welch’s t test when variances were unequal), and the Mann–Whitney U test otherwise; group means are reported descriptively.

Comparisons of spermatological and kinematic parameters between production types were performed using an independent samples t test when assumptions were satisfied, and the Mann–Whitney U test otherwise. For these outcomes, the unit of analysis was the bull; for each bull, parameters were summarised as the mean of repeated straw measurements (six straws per bull), and between-group tests were conducted on these bull-level means (beef *n* = 5; dairy *n* = 6).

Pregnancy outcome (pregnant vs. non-pregnant) was compared between groups using the chi-square test, with Fisher’s exact test applied when expected cell counts were <5. Pregnancy outcome comparisons were performed at the insemination level; clustering by bull was not modelled in these univariate tests. Mean bull-level pregnancy rates were additionally summarised descriptively by production type.

Associations between semen quality variables and fertility were evaluated using Pearson’s product–moment correlation. Semen parameters were summarised as bull-level means derived from repeated straw measurements, and fertility was expressed as the bull-level pregnancy rate calculated from inseminations with a valid pregnancy diagnosis. Pearson correlation coefficients (*r*) were computed between pregnancy rate and motility, progressive motility, concentration, VAP, VSL, VCL, STR, LIN, WOB, ALH, BCF, separately within beef bulls (*n* = 5; 190 inseminations with valid pregnancy outcome) and dairy bulls (*n* = 6; 219 inseminations with valid pregnancy outcome). Linearity and influential outliers were assessed visually. Two-sided *p* values were reported and statistical significance was set at *p* < 0.05; p values were reported to four decimal places when appropriate, and very small values as *p* < 0.0001. No adjustment for multiple comparisons was applied.

## 3 Results

### 3.1 Insemination counts per bull

**Figure 2:**
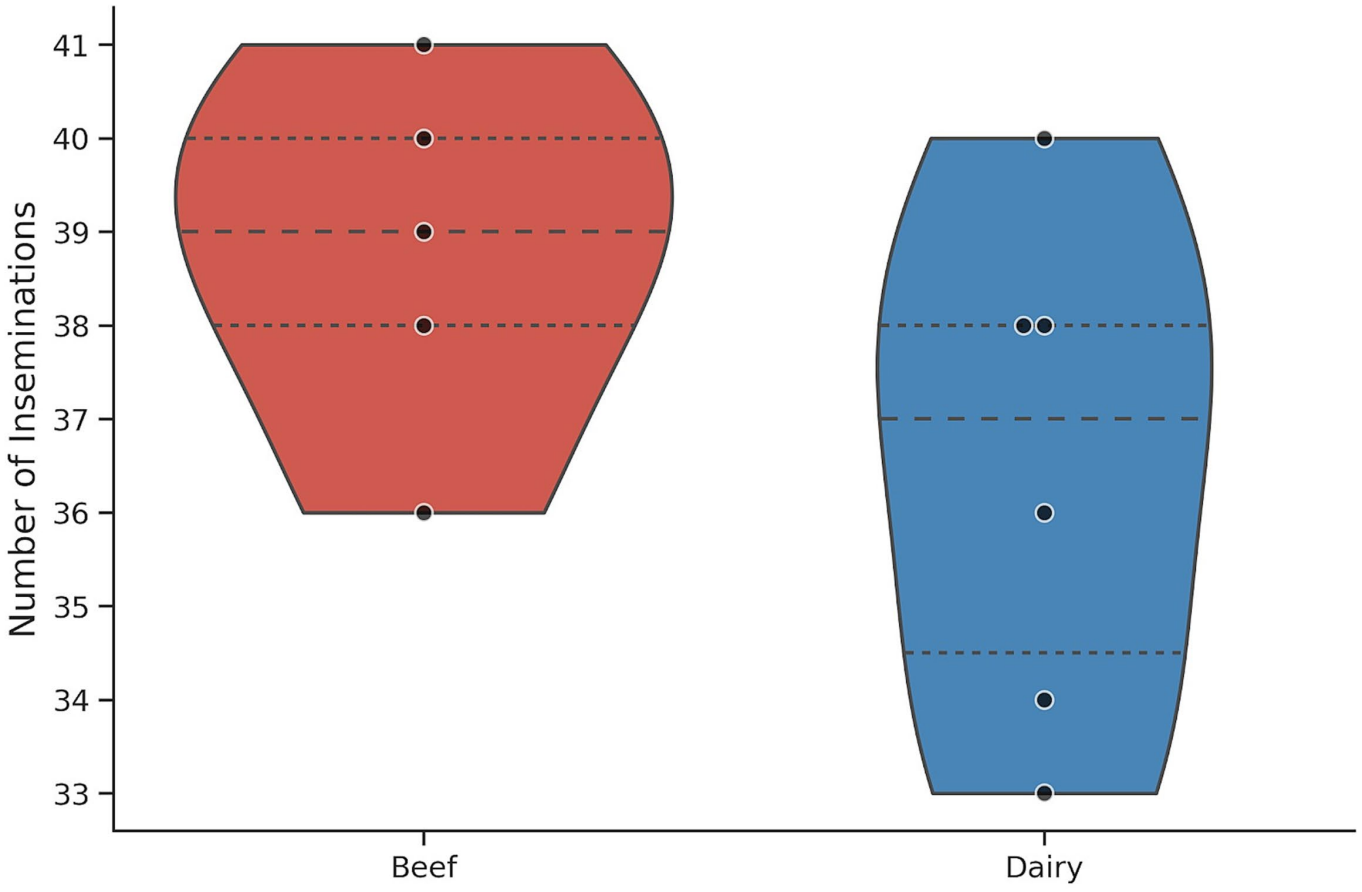
Distribution of insemination counts per bull in beef and dairy bulls. It shows the distribution of the number of inseminations per bull by production type. Dots indicate individual bulls; the violin width represents kernel density, and the dashed lines indicate the median and interquartile range. Counts reflect total inseminations performed per bull (beef total = 194; dairy total = 219); pregnancy-rate analyses were restricted to inseminations with a valid pregnancy diagnosis (beef = 190; dairy = 219).

Insemination counts per bull were examined within each production type (Beef: *n* = 194 inseminations; Dairy: *n* = 219 inseminations; Figure 2). Bull-level insemination counts showed no evidence of departure from normality in either group (Beef: W = 0.979, *p* = 0.928; Dairy: W = 0.948, *p* = 0.721), and variances were comparable between groups (Levene’s test, *p* = 0.337).

### 3.2 Spermatological findings

Semen from beef bulls showed higher total motility than semen from dairy bulls (83.59 ± 2.08% vs. 56.24 ± 2.42%; *p* < 0.0001) (Table 1). Progressive motility was also higher in beef bulls (58.54 ± 3.01% vs. 48.25 ± 9.57%; *p* = 0.0475) (Table 1). Sperm concentration did not differ significantly between groups (in beef bulls and dairy bulls repectively; 29.93 ± 6.70 vs. 26.73 ± 2.28 × 10^6^/mL; *p* = 0.2970) (Table 1).

**Table 1:** Comparison of sperm kinematic parameters between beef and dairy bulls.

Parameters	Beef	Dairy	*p*-value	Significance
Motility (%)	83.59 ± 2.08	56.24 ± 2.42	<0.0001	***
Progressive motility (%)	58.54 ± 3.01	48.25 ± 9.57	0.0475	*
Sperm concentration (10^6^/mL)	29.93 ± 6.70	26.73 ± 2.28	0.2970	ns
VAP (μm/s)	59.06 ± 11.02	75.24 ± 14.94	0.0762	ns
VSL (μm/s)	46.73 ± 5.42	53.33 ± 4.93	0.0635	ns
VCL (μm/s)	78.09 ± 5.49	80.94 ± 5.74	0.4248	ns
STR (%)	65.93 ± 3.13	70.18 ± 2.51	0.0336	*
LIN (%)	47.22 ± 1.44	55.99 ± 4.69	0.0031	**
WOB (%)	68.42 ± 4.00	73.02 ± 3.03	0.0582	ns
ALH (μm)	3.19 ± 0.23	2.64 ± 0.26	0.0055	**
BCF (Hz)	7.46 ± 0.18	7.12 ± 0.28	0.0821	ns

Values are presented as mean ± SD, ns, not significant (*p* > 0.05),*p < 0.05, **p < 0.01, ***p < 0.001.

For velocity descriptors, dairy bulls had numerically higher VAP (75.24 ± 14.94 vs. 59.06 ± 11.02 μm/s; *p* = 0.0762) and VSL (53.33 ± 4.93 vs. 46.73 ± 5.42 μm/s; *p* = 0.0635), whereas VCL was similar dairy and beef bulls (80.94 ± 5.74 vs. 78.09 ± 5.49 μm/s; *p* = 0.4248) (Table 1). STR and LIN were higher in dairy bulls (STR: 70.18 ± 2.51 vs. 65.93 ± 3.13%, *p* = 0.0336; LIN: 55.99 ± 4.69 vs. 47.22 ± 1.44%, *p* = 0.0031), while ALH was lower in dairy bulls (2.64 ± 0.26 vs. 3.19 ± 0.23 μm; *p* = 0.0055) (Table 1). WOB and BCF did not differ significantly between dairy and beef bulls (WOB: *p* = 0.0582; BCF: *p* = 0.0821) (Table 1).

### 3.3 Daily milk yield

Milk yield differed markedly between production systems, with dairy cows producing substantially higher daily milk yields (18.47 ± 0.38 kg/d; *n* = 219) compared with beef cows (6.21 ± 0.19 kg/d; *n* = 194; *p* < 0.001).

### 3.4 Fertility findings

**Figure 3:**
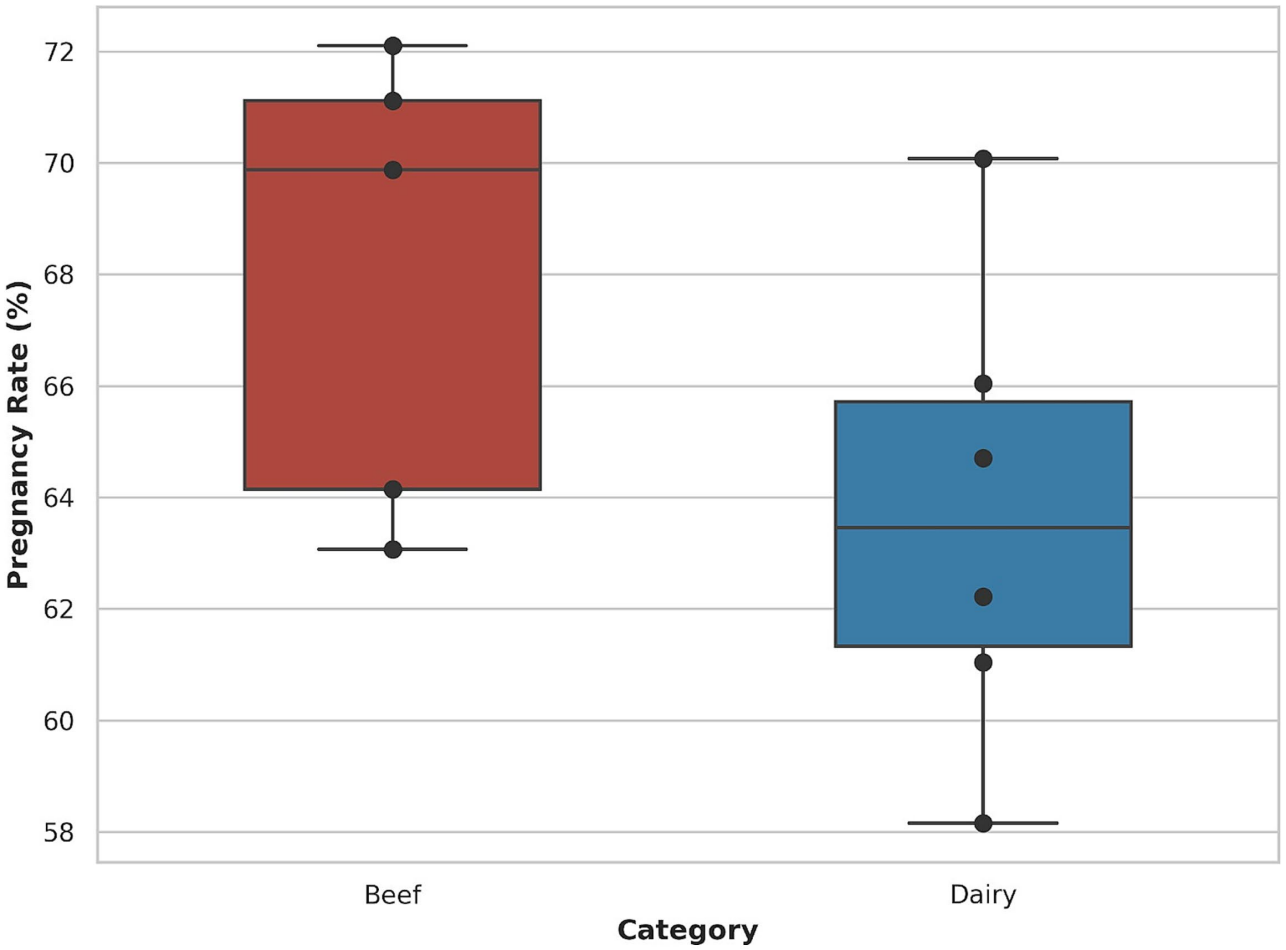
Bull-level pregnancy rates following inseminations using semen from beef and dairy bulls.

Pregnancy outcomes showed numerical differences between production types (Figure 3). The mean bull-level pregnancy rate was 68.06% for beef bulls and 63.71% for dairy bulls. The between-group comparison of pregnancy outcome at the insemination level did not reach statistical significance (*p* > 0.05).

### 3.5 Correlation between fertility and spermatological parameters

**Figure 4:**
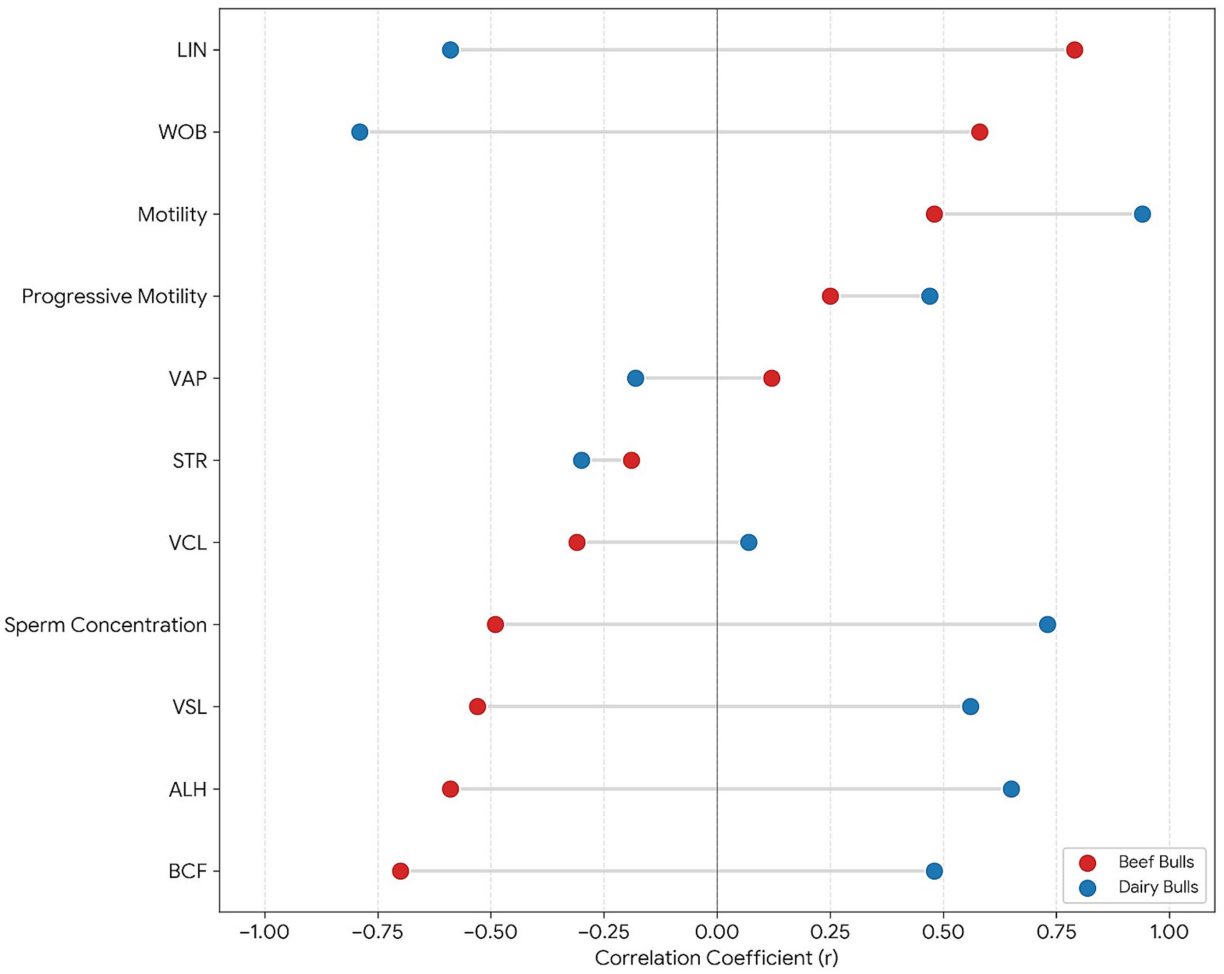
Within-group Pearson correlations between bull-level semen kinematic parameters and pregnancy rate in dairy and beef bulls.

Within-group correlation patterns were evaluated separately for beef bulls (190 inseminations with valid pregnancy outcome) and dairy bulls (219 inseminations with valid pregnancy outcome). Figure 4 presents within-group Pearson correlation coefficients (*r*) for the selected semen parameters. Motility showed positive correlations with pregnancy rate in both groups (beef: *r* = 0.48; dairy: *r* = 0.94). Correlation directions differed between production types for LIN (beef: *r* = 0.79; dairy: *r* = −0.59), WOB (beef: *r* = 0.58; dairy: *r* = −0.79), ALH (beef: *r* = −0.59; dairy: *r* = 0.65), BCF (beef: *r* = −0.70; dairy: *r* = 0.48), and sperm concentration (beef: *r* = −0.49; dairy: *r* = 0.73). Correlation coefficients should be interpreted cautiously due to the limited number of bulls per group.

## 4 Discussion

In the present study, spermatological characteristics of frozen–thawed semen and field fertility outcomes were evaluated in dairy and beef bulls under commercial insemination conditions. The most marked between-group difference was observed in motility, with semen from beef bulls showing substantially higher total motility and higher progressive motility than semen from dairy bulls. In contrast, the kinematic profile did not indicate a consistent advantage of one production type across velocity descriptors, and several velocity measures showed limited separation between groups. This pattern is compatible with previous reports suggesting that individual CASA-derived kinematic variables often have restricted predictive value for field fertility when considered in isolation, particularly in multifactorial commercial settings (3, 14).

Interpretation of semen type effects on fertility in this dataset needs to be made in the context of the female populations in which inseminations occurred. Semen from dairy bulls was used in high-yield dairy females, whereas semen from beef bulls was used in beef females, and the two female groups differed markedly in daily milk production (18.47 ± 0.38 vs. 6.21 ± 0.19 kg/d). Given the well-described association between high milk yield and reduced reproductive efficiency in dairy cattle, the lower pregnancy performance observed in the dairy female group could plausibly reflect female-side physiological constraints, including a higher likelihood of negative energy balance, compromised follicular dynamics, and altered uterine conditions that may reduce early embryo survival, even when semen meets acceptable laboratory quality thresholds (15–17). Under this structure, differences in pregnancy outcomes should not be interpreted as evidence that CASA kinematics are biologically irrelevant; rather, they underscore that field fertility is jointly determined by semen characteristics and the metabolic and management context of the female, and that the latter may dominate the observable outcome in high-producing systems.

This framing is consistent with large-scale evidence for substantial between-bull variation in field fertility under broadly comparable management conditions (18), while acknowledging that the magnitude of any bull effect can be attenuated when conception is primarily constrained by female-related factors linked to production intensity. In practical terms, the coupling of semen type with female production type in the present field setting represents a potential source of confounding and should be considered when attributing differences in pregnancy outcomes to bull production type alone. Accordingly, these comparisons are observational and do not support causal attribution of pregnancy differences to bull production type.

A further observation concerned the dispersion of selected kinematic and spermatological measures across bulls within each production type. In this dataset, variability appeared more pronounced for some traits in dairy bulls, most notably for progressive motility, whereas other parameters showed comparable dispersion between groups. Any interpretation of between-bull variability should therefore remain trait-specific rather than assuming a uniform pattern across all CASA outputs. Differences in within-group dispersion could plausibly relate to population structure and breeding history, including differences in effective population size, the intensity and direction of selection, and the extent to which reproductive traits have been directly or indirectly targeted in breeding programs. Dairy populations, particularly Holstein, have been shaped by multi-trait selection indices that integrate production, health, and efficiency traits, while beef populations are often managed under different breeding objectives and selection pipelines. Such contrasts may contribute to heterogeneity in sperm phenotypes, although the present data do not allow causal attribution. Prior CASA-based comparisons across breeds and production types provide context for observed differences in sperm motion characteristics (19, 20), but the extent to which they explain within-type variability requires dedicated analyses.

To reduce cross-system heterogeneity, correlations between semen kinematics and fertility were examined within production type rather than in a pooled dataset. Pooling dairy and beef systems would combine semen measurements with substantially different female production contexts, which could introduce structural confounding and obscure kinematic-fertility relationships. The within-group approach therefore provides a more interpretable framework for relating variation in kinematic parameters to pregnancy rate within a given production setting.

Within this within-group framework, motility showed the most consistent positive association with fertility in both production types, although the strength differed. In beef bulls (*n* = 5; 190 inseminations with valid pregnancy outcome), pregnancy rate correlated positively with motility (*r* = 0.48). In dairy bulls (*n* = 6; 219 inseminations with valid pregnancy outcome), the corresponding association was stronger (*r* = 0.94). Beyond motility, several kinematic descriptors displayed marked direction changes between production types. Linearity (LIN) and wobble (WOB) were positively correlated with fertility in beef bulls (LIN *r* = 0.79; WOB *r* = 0.58) but negatively correlated in dairy bulls (LIN *r* = −0.59; WOB *r* = −0.79). Conversely, ALH and BCF showed negative correlations with fertility in beef bulls (ALH *r* = −0.59; BCF *r* = −0.70) and positive correlations in dairy bulls (ALH *r* = 0.65; BCF *r* = 0.48). Sperm concentration followed the same pattern of sign reversal, with a negative association in beef bulls (*r* = −0.49) and a stronger positive association in dairy bulls (*r* = 0.73). These within-type patterns indicate that while motility aligned with fertility in both contexts, several ratio-derived trajectory and beat-frequency descriptors were associated with fertility in production-type-specific directions.

The production-type-specific sign reversals observed for LIN, WOB, ALH, BCF, and concentration suggest that the fertility relevance of individual CASA descriptors may be context dependent rather than universal. Several of these indices are mathematically coupled or ratio derived, and they can reflect different underlying motion phenotypes depending on the distribution of velocity components, tracking settings, and the biological state of the sperm population at assessment. Within-group correlations were estimated on a small number of bulls, making coefficients sensitive to influential observations and limiting the strength of inference. For these reasons, the within-type association structure is best interpreted as exploratory evidence that the mapping between kinematic phenotypes and field fertility may differ between production contexts, rather than as definitive proof of opposite causal mechanisms.

Velocity descriptors are often assumed to be beneficial proxies for sperm performance, yet field data do not consistently support this assumption. In the present study, velocity-related CASA measures did not provide a stable or production-type-robust fertility signal, and interpretation of these descriptors therefore warrants caution. One mechanistic explanation that remains plausible, although it was not directly evaluated here, is that elevated velocity shortly after thawing can coincide with cryo-induced signalling changes that reduce functional longevity. Premature capacitation, often termed cryo-capacitation, has been described in frozen–thawed bovine semen and involves membrane and signalling alterations that resemble physiological capacitation (21). Under this framework, a subpopulation may display hyperactivation-like movement soon after thawing, which can appear favourable in CASA snapshots but may be coupled to accelerated energy expenditure and a shorter functional lifespan within the female reproductive tract. Hyperactivation-like motility has also been linked to increased metabolic demand and reactive oxygen species generation, which may exacerbate oxidative stress and compromise membrane integrity and chromatin stability (22). These mechanisms provide biological context for why velocity-related CASA metrics may not map straightforwardly onto field pregnancy outcomes.

More broadly, these results indicate that production type related differences in semen characteristics are not necessarily mirrored by proportional differences in field fertility. Conception in commercial systems reflects a multifactorial process in which semen attributes interact with female physiological status, estrus detection and insemination timing, environmental conditions, and herd level management. Accordingly, favourable laboratory-derived semen metrics, even when they capture clear contrasts between bull groups, may be insufficient on their own to ensure high conception rates under field conditions.

Several limitations should be acknowledged. First, although a total of 413 inseminations were performed, pregnancy outcome was available for 409 inseminations, and the number of bulls contributing semen to the study was limited (*n* = 11). This constrains inference at the bull level and warrants confirmation in larger and more diverse bull populations, particularly for within-type association analyses. Second, inseminations were carried out under typical farm conditions, with semen from dairy bulls used in dairy females and semen from beef bulls used in beef females. As a result, female production type and physiological status could not be experimentally standardized, and residual confounding by female-related factors cannot be excluded. At the same time, this structure reflects routine breeding practice and increases ecological validity. Differences between female groups were quantified using milk yield records and were considered when interpreting fertility outcomes in relation to semen characteristics. Because the production-type contrast also corresponds to a breed contrast in the present dataset, generalisation to beef and dairy bulls more broadly should be made cautiously. Given the field-based design, the study is subject to structural confounding because semen type was not assigned independently of the female production context. Beef-bull semen was used in beef females and dairy-bull semen in high-yielding dairy females, coupling semen type with female physiology and herd-level management conditions. Consequently, pregnancy-rate differences cannot be experimentally attributed to semen characteristics alone, and the findings should be interpreted as context-dependent associations under commercial conditions rather than causal effects of bull production type.

Future studies in larger bull cohorts could apply hierarchical modelling frameworks to quantify the independent contributions of semen traits and female-side factors to pregnancy outcome.

Taken together, the present findings suggest that the association structure between CASA-derived kinematic descriptors and fertility can differ by production context, while motility remains a consistently informative correlate within both beef and dairy bulls. Practical evaluation of frozen semen is therefore likely to benefit from multidimensional assessment strategies that integrate kinematic profiles with broader functional indicators and that explicitly consider the physiological and management context of the inseminated females.

## Data Availability

The original contributions presented in the study are included in the article/supplementary material, further inquiries can be directed to the corresponding author.

## References

[1] Baruselli PS Ferreira RM Sá Filho MFD Bó GA. Using artificial insemination vs. natural service in beef herds. Animal. (2018) 12:s45–52. doi: 10.1017/S175173111800054X 29554986

[2] Netherton JK Robinson BR Ogle RA Gunn A Villaverde AISB Colyvas K . Seasonal variation in bull semen quality demonstrates there are heat-sensitive and heat-tolerant bulls. Sci Rep. (2022) 12:15322. doi: 10.1038/s41598-022-17708-9, 36097009 PMC9468146

[3] Nagy S Polichronopoulos T Gábor G Solti L Cseh S. Correlation between bull fertility and sperm cell velocity parameters generated by computer-assisted semen analysis. Acta Vet Hung. (2015) 63:370–81. doi: 10.1556/004.2015.035, 26551427

[4] Khan I Mesalam A Heo YS Lee SH Nabi G Kong IK. Heat stress as a barrier to successful reproduction and potential alleviation strategies in cattle. Animals. (2023) 13:2359. doi: 10.3390/ani13142359, 37508136 PMC10376617

[5] Holt WV. Fundamental aspects of sperm cryobiology: the importance of species and individual differences. Theriogenology. (2000) 53:47–58. doi: 10.1016/s0093-691x(99)00239-3, 10735061

[6] Azevedo HC Blackburn HD Lozada-Soto EA Spiller SF Purdy PH. Enhancing evaluation of bull fertility through multivariate analysis of sperm. J Anim Sci. (2024) 107:11774–84. doi: 10.3168/jds.2024-25163, 39343204

[7] Brito LFC Silva AEDF Rodrigues LH Vieira FV Deragon LAG Kastelic JP. Effects of environmental factors, age and genotype on sperm production and semen quality in Bos indicus and *Bos taurus* AI bulls in Brazil. Anim Reprod Sci. (2002) 70:181–90. doi: 10.1016/s0378-4320(02)00009-x, 11943488

[8] Morrell JM Valeanu AS Lundeheim N Johannisson A. Sperm quality in frozen beef and dairy bull semen. Acta Vet Scand. (2018) 60:41. doi: 10.1186/s13028-018-0396-2, 29973236 PMC6031104

[9] Hoflack G Opsomer G Van Soom A Maes D de Kruif A Duchateau L. Comparison of sperm quality of Belgian blue and Holstein Friesian bulls. Theriogenology. (2006) 66:1834–46. doi: 10.1016/j.theriogenology.2006.05.007, 16815541

[10] Dönmez H İnanç ME. Spermatological parameters in vitro of Holstein bulls and relationship with male fertility. Acta Sci Vet. (2024) 52:1–8. doi: 10.22456/1679-9216.140919

[11] İnanç ME Cil B Tekin K Alemdar H Daşkin A. The combination of CASA kinetic parameters and fluorescein staining as a fertility tool in cryopreserved bull semen. Turk J Vet Anim Sci. (2018) 42:452–8. doi: 10.3906/vet-1801-83

[12] Hitit M Ugur MR Dinh TTN Sajeev D Kaya A Topper E . Cellular and functional physiopathology of bull sperm with altered sperm freezability. Front Vet Sci. (2020) 7:581137. doi: 10.3389/fvets.2020.581137, 33195596 PMC7644894

[13] Amann RP Waberski D. Computer-assisted sperm analysis (CASA): capabilities and potential developments. Theriogenology. (2014) 81:5–17. doi: 10.1016/j.theriogenology.2013.09.004, 24274405

[14] Gil J Januskauskas A Håård M Håård MGM Johanisson A Söderquist L . Functional sperm parameters and fertility of bull semen extended in Biociphos-plus® and Triladyl®. Reprod Domest Anim. (2000) 35:69–77. doi: 10.1046/j.1439-0531.2000.00197.x

[15] Kasalak Y İnanç ME. Interrelationships between milk yield, anti-müllerian hormone levels, metabolic profile parameters, and first service conception rates in Simmental cows. Ankara Üniv Vet Fak Derg. (2025) 72:487–96. doi: 10.33988/auvfd.1640250

[16] Civiero M Cabezas-Garcia EH Ribeiro-Filho HMN Gordon AW Ferris CP. Relationships between energy balance during early lactation and cow performance, blood metabolites, and fertility: a meta-analysis of individual cow data. J Dairy Sci. (2021) 104:7233–51. doi: 10.3168/jds.2020-19607, 33685685

[17] Martens H. Invited review: increasing milk yield and negative energy balance: a gordian knot for dairy cows? Animals. (2023) 13:3097. doi: 10.3390/ani13193097, 37835703 PMC10571806

[18] Berry DP Evans RD McParland S. Evaluation of bull fertility in dairy and beef cattle using cow field data. Theriogenology. (2011) 75:172–81. doi: 10.1016/j.theriogenology.2010.08.002, 20875673

[19] Hoflack G Opsomer G Rijsselaere T Van Soom A Maes D De Kruif A . Comparison of computer-assisted sperm motility analysis parameters in semen from belgian blue and Holstein–Friesian bulls. Reprod Domest Anim. (2007) 42:153–61. doi: 10.1111/j.1439-0531.2006.00745.x, 17348972

[20] Cole JB Wiggans GR Ma L Sonstegard TS Lawlor TJ Jr Crooker BA . Genome-wide association analysis of thirty-one production, health, reproduction and body conformation traits in contemporary US Holstein cows. BMC Genomics. (2011) 12:408. doi: 10.1186/1471-2164-12-408 21831322 PMC3176260

[21] Bailey JL Bilodeau JF Cormier N. Semen cryopreservation in domestic animals: a damaging and capacitating phenomenon. J Androl. (2000) 21:1–7. doi: 10.1002/j.1939-4640.2000.tb03268.x 10670514

[22] Bollwein H Bittner L. Impacts of oxidative stress on bovine sperm function and subsequent in vitro embryo development. Anim Reprod. (2018) 15:703–10. doi: 10.21451/1984-3143-AR2018-0041, 36249836 PMC9536048

